# Life-Changing Experiences of Mothers with School-Age Children during the COVID-19 Pandemic: Focusing on Their Health Risk Perceptions and Health-Related Behaviors

**DOI:** 10.3390/ijerph18094523

**Published:** 2021-04-24

**Authors:** Hye Jin Yoo, JaeLan Shim, Namhee Kim

**Affiliations:** 1 College of Nursing, Dankook University, Cheonan 31116, Korea; yoohj21@dankook.ac.kr; 2 Department of Nursing, Dongguk University, Gyeongju 380660, Korea; 3 College of Nursing, Brain Korea 21 FOUR Project, Yonsei University, Seoul 03722, Korea; namheekim0316@gmail.com

**Keywords:** COVID-19, epidemics, health behavior, health risk appraisal, pandemics, qualitative research

## Abstract

This study aimed to explore health risk perceptions, changes in health-related behaviors, and life experiences of mothers with school-age children during the early coronavirus disease (COVID-19) pandemic. Data were collected between 16 July and 10 September 2020, by individual interviews and analyzed through qualitative content analysis. After the twelve participants’ experiences were analyzed, four themes and ten sub-themes were derived. The four themes were: “Struggling to identify the substance of COVID-19,” “Taking the initiative to protect the health of the family,” “Frustrated by the brutal reality of no end in sight,” and “Trying to adjust wisely to an inevitable new lifestyle.” The findings suggest that while the world remains in an ongoing battle with COVID-19, national health institutions should prepare a health education system for specific infection prevention methods that can be practiced by individuals in daily life.

## 1. Introduction

In Korea, when a Chinese tourist was diagnosed as the first confirmed case of COVID-19 on 20 January 2020, the government raised the country’s COVID-19 crisis alert level from “attention” to “caution” and established an emergency response committee called the Central Disaster and Safety Countermeasure Headquarters. Korea also declared certain regions of the country as special disaster zones and began to implement a code of conduct for the public and take preventive measures against the spread of COVID-19 [1,2].

In response to the increasing spread of COVID-19, the Korean government implemented an intensive social distancing policy beginning March 2020 that constitutes government-recommended regulations to prevent and block any community spread of the virus [1]. The relevant guidelines include avoiding social events and gatherings, staying at home, expanding the ability to work from home, and avoiding crowded places, in addition to basic preventive measures, such as washing one’s hands with soap and running water and wearing a mask when outdoors. The guidelines are categorized into three levels [3,4,5] and enforced according to the severity of the spread of COVID-19, as well as the strength of the preventive measures that have been established [1].

The COVID-19 pandemic has brought about sudden and significant changes to people’s everyday lives, including the imposed isolation of individuals internationally, illnesses caused by the virus, loss of employment, and financial burdens. These changes present factors that could significantly affect families [6]. In addition, it is likely that these changes have had a considerable impact on peoples’ personal health risk perceptions and, further, an effect on the health-related behaviors of families. In particular, it is understood that determining the COVID-19 related experiences of mothers with school-age children—who are not capable of complete self-regulation and are therefore vulnerable to group infections—is significant. These mothers can offer various perspectives not only on raising and educating children but also about their health risk perceptions, health management, and health-related behaviors concerning their family’s health as well as their own.

The study objective was to examine the health risk perceptions and experiences with health-related behaviors during the spread of COVID-19 by conducting in-depth investigations of the experiences of mothers with school-age children. This study aimed to specifically determine health risk perceptions and changes in health-related behaviors (including the process of those changes), as well as the life experiences and their meanings among mothers of school-age children during the ongoing COVID-19 pandemic. Further, this study provides preliminary data for the development of an effective family-based health promotion intervention. The data will contribute to establishing a detailed foundation for policies and measures aimed at mitigating the effects of COVID-19, as well as confronting potential future pandemics caused by unseen infectious agents.

## 2. Materials and Methods

### 2.1. Study Design

**Figure 1:**
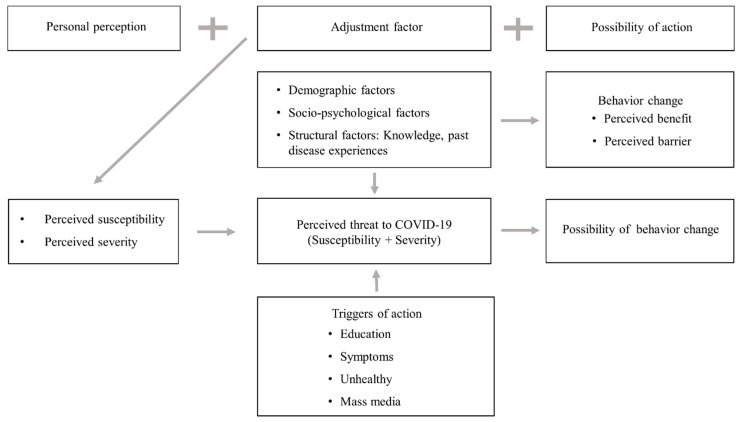
The health belief model, which is the applied model of this study.

This study applied a qualitative research design to understand the health risk perceptions, changes in health-related behaviors, and life-changing experiences of mothers with school-age children during the home isolation period of social distancing measures during the COVID-19 pandemic. In this study, the health belief model [7] was applied as a theory to explain the transitional type of health behavior exhibited during the COVID-19 pandemic (Figure 1).

The health belief model [7] is widely known as a theoretical framework developed to explain how preventive health behaviors are performed under certain conditions. In this study, the perceived susceptibility, which is how sensitive a person will be to the disease without treatment, influences the perceived threat of COVID-19, and this perceived threat turns into preventive health behavior through education, symptoms, unhealthy conditions, and spills of information.

### 2.2. Participants

The participants of this study were women with school-age children who fully understood the purpose of the research and voluntarily agreed to participate. The inclusion criteria for participants comprised the following: (a) mothers with school-age children, (b) a woman who lives with her husband and child to form a family, and (c) ability to communicate in Korean. Participants were recruited via convenience sampling and snowball sampling. First, we selected one participant with characteristics suitable for the study topic and inclusion criteria. Then, we selected participants based on the recommendation of the sampled participant.

A purposive snowball sampling method was used, in which participants who had ample research-related experiences to report recruited others, resulting in a total of twelve participants. All the participants were women aged 36–45 years, with an average age of 40.42 (SD = 3.06) (Table 1).

### 2.3. Data Collection

Data collection took place between 16 July and 10 September 2020. The interviews were conducted individually as single sessions. While the participants were able to choose a comfortable location for the interview, they all chose to be interviewed at their respective homes. The interviews lasted approximately 60–90 min each and were recorded with participants’ consent. The principal research question was, “What were your health risk perceptions and experiences with regards to health-related behavior changes during COVID-19?” A semi-structured questionnaire was used during each interview. To develop the interview questions, the researchers explained the purpose of this research to a nursing professor and three parents who had PhDs in nursing and gathered their opinions to compose the questions, which were also based on those from a previous study [3] (Table 2).

### 2.4. Ethical Considerations and Investigator Training and Preparation

The data were encoded to prevent the disclosure of any personal information, and efforts were made to ensure the anonymity of the study participants. The text of the collected handwritten forms was analyzed, with all of the personally identifiable information erased.

The researchers completed courses on qualitative research methods and interview training through continuous meetings with a qualitative research specialist, which provided them numerous experiences in conducting qualitative interviews and data analysis prior to the study.

### 2.5. Data Analysis

This study used inductive content analysis to analyze health risk perceptions, changes in health-related behaviors, and life-changing experiences of mothers with school-age children during the COVID-19 pandemic [8]. The inductive content analysis method is used when existing theories about the phenomena to be studied are limited or there are insufficient considerations. Rather than naming categories from existing data, the researcher directly derived meaning from the data of the current study through new insights. This is an inductive method [8].

First, in the preparatory stage, directions for the approach, unit of analysis, and sense of data were determined using Microsoft Excel. In the next organizational stage, the researcher repetitively read and became immersed in the data without excluding theoretical bases or general knowledge. Patterns and subjects of phrases and sentences spoken by all participants were found and coded (open coding). Then, reading the coded material again, the researcher grouped similar concepts together. Based on this, after categorization of semantic units and consideration of their commonalities and relevances, semantic units were abstracted for each subject, and the core subject was then derived. At this time, the categorized data were continually checked for consistency with the actual participants’ statements. In the final step, the main themes that had been derived were defined and described by the participants’ experiences for each sub-area. 

### 2.6. Research Rigor

To ensure the precision of this qualitative research, this study aimed to increase the credibility, fittingness, auditability, and confirmability of the research, according to the evaluation criteria proposed by Sandelowski [9]. In order to increase credibility, the interviews were conducted in a quiet place, and consideration was made so that a genuine story could be related. To ensure the reliability of analysis and interpretation, feedback from qualitative researchers was collected, and three participants were verified. Participants who could explain the phenomena experienced for fitness were selected, and data collection and analysis were conducted until reaching a point when new content was no longer available. For auditability, original data on topics derived from research results were provided so that readers could understand the decision-making process from data to results. For confirmability, bias or prejudice on the participant’s statement was constantly checked, and the prejudice of the researcher was minimized to maintain neutrality.

## 3. Results

As a result of analyzing the experiences of the twelve participants, a total of 29 codes were composed. Ten sub-themes with a more abstract and comprehensive meaning were extracted from these codes. The ten sub-themes were combined into four themes that best described the life-changing experiences of mothers with school-age children during the spread of COVID-19, with a focus on health perceptions and health-related behaviors (Table 3).

### 3.1. Theme 1: Struggling to Identify the Substance of COVID-19

Inaccurate information included in the daily influx of information caused more confusion for participants in determining how to deal with COVID-19; they were desperate for accurate information to protect their families from infection. The participants did their best to obtain accurate information regarding COVID-19 and infection prevention through social network services (SNS), television news programs, online news articles, and via SNS conversations with both acquaintances and healthcare providers.

#### 3.1.1. Floundering in a Deep Swamp of Confusion and Fear

The participants felt increasing terror and fear as they obtained COVID-19 related information on a daily basis from diverse media sources through a variety of communication devices. The excessive amount of COVID-19 related information caused considerable confusion for the participants.

“Coronavirus-related news, media, Internet, cell phone text messages on a daily basis… I felt confused by the constant stream of information saying this is right, or that is right. If there’s news of a confirmed case in the vicinity, I feel hectic from searching the patient’s path of movement.”

(Participant 1)

As the participants learned about COVID-19 related information, their fears grew. They especially felt threatened by the news of confirmed cases in their respective neighborhoods and were worried that they or their families were not safe, with the COVID-19 situation being perceived as terrifying.

“When there was news that a patient—of which I’d only heard of over the news—had visited my neighborhood, there was a feeling of panic in a chat room I was in with a group of moms. The thought that this virus is a conspiracy even entered my mind and I felt scared and terrified.”

(Participant 2)

#### 3.1.2. Desperately Wanting Reliable Information

As the spread of COVID-19 infections worsened, the participants wanted accurate health-related information for the prevention of this illness, in addition to the abundance of information appearing on the news daily, such as the number of confirmed cases, paths of patients’ movements, and times of occurrence. They felt uneasy about being in a situation wherein they could only obtain information by word of mouth.

“The information released on the news…is informative, but there’s insufficient information for the prevention of the coronavirus. As there wasn’t accurate information regarding the prevention of infection, I began to rely on Internet searches, acquaintances who are doctors, and a chat room with a group of moms.”

(Participant 5)

### 3.2. Theme 2: Taking the Initiative to Protect the Health of the Family

As COVID-19 became more prevalent internationally, the participants worked hard to prevent infections within their families. In order to do their best for their family’s health, they taught their relatives the basic methods of preventing infection, such as wearing masks, practicing regular hand washing, and so on. They also had their family consume immune-boosting foods, dietary supplements, functional foods, etc., with a portion of participants trying methods of symptomatic therapy that had not been proven as medically effective.

#### 3.2.1. Devised Methods for the Prevention of Infections

The participants faced difficulties in obtaining masks and hand sanitizers—the basic materials needed for the prevention of COVID-19 infection. They would stand in lines outside pharmacies or stores in the early hours of the morning to purchase masks and would repeatedly search online and visit multiple places before finally being able to purchase them.

“I visited ten pharmacies to purchase masks and hand sanitizers. I even waited in line for masks since two o’clock in the morning before buying them. It was the first time in my life that I had waited in line to buy masks.”

(Participant 1)

Furthermore, they used a variety of methods to protect their family from infection, such as prohibiting their relatives from leaving the house, getting them to use disposable gloves when leaving the house, disinfecting all items, etc.

“I didn’t leave the house with my kids for almost two months because I was anxious. And then, when we’d leave the house, I was busy getting masks, disposable gloves, and a spray bottle with an alcohol solution.”

(Participant 9)

#### 3.2.2. Focus on Family’s Health Management

The participants thought that, while being cautious of COVID-19 and effectively enduring this pandemic, it was important to live a healthy lifestyle. They expressed a sense of responsibility as the person in charge of protecting their family’s health and, together with their husbands, worked actively inside and outside of the household to protect it.

“I moved quickly to buy products or foods that were known to be good for health. I really put a lot of effort into feeding my children—who were home all day—good quality food and taking them outside every day when there were few people out so they could get even a little bit of exercise. I think—as a mother and a wife—my sense of duty to protect my family was aroused.”

(Participant 1)

#### 3.2.3. Attempted Symptomatic Therapy

Due to the insufficient amount of information regarding infection prevention, the participants had no choice but to rely on symptomatic therapy methods that had not been proven as medically effective. They attempted any method that was rumored to be effective in strengthening one’s immune system.

“I tried anything that was known to be good for health. When I heard that Betadine sprays were effective in treating sore throats, I sprayed my children’s throats twice a day. I had also heard that brushing teeth with salt water was good for immunity, so I did that with my kids and husband every day.”

(Participant 6)

### 3.3. Theme 3: Frustrated by the Brutal Reality of No End in Sight

As the spread of COVID-19 continued, the participants gradually experienced fatigue. Contrary to expectations that the pandemic would end in a few months, the subsequent unpredictability of its endpoint caused participants to feel frustrated and become increasingly stressed.

#### 3.3.1. Dissatisfaction with a Lifestyle That Has No Way Out

The participants expressed physical and mental difficulties enduring the changes in their everyday lives caused by COVID-19 regulations.

“I thought this wouldn’t last for very long but as the coronavirus situation is lengthening, I feel frustrated and annoyed. I don’t know how much longer I’d have to live like this, with all activities put on hold; it’s like running through a tunnel without a visible exit.”

(Participant 5)

They wondered when this situation would end. As the fear that they may have to continue living this way grew among them, they began to wish to return to their previous everyday lives.

“I am worried we won’t be able to return to the time before the coronavirus. Honestly, I thought it would end after a month or two. I feel like I’m going crazy. If I knew when it would end, I’d feel encouraged.”

(Participant 7)

#### 3.3.2. Yearning for People from within a Dreary Reality

As they had to wear masks and avoid physical encounters with people, the participants missed their past daily routines when their children would attend school every day, and they would freely meet and socialize with others without wearing a mask.

“My children used to go to the library often and read books, but now—without meeting people—we request the books in advance using a computer, and we use a system of borrowing and returning books through a machine. It’s dreary and sad that the everyday lives we took for granted have all changed, so that encounters with people are disappearing.”

(Participant 1)

The participants became aware of the importance of human relationships in their lives and realized the positive emotional effect of human interactions.

“I miss people. Will the day really come again when we are able to take off our masks and talk to people face-to-face?”

(Participant 3)

### 3.4. Theme 4: Trying to Adjust Wisely to an Inevitable New Lifestyle

The participants correctly perceived the nature of the COVID-19 situation and began to move on, from missing their past lives to adjusting to new lifestyles altered by this pandemic.

#### 3.4.1. Strengthened Family Bonds

As time spent with their families grew, the participants experienced a growing sense of familial love through sharing leisure activities and hobbies. As the communal sense of “us” became stronger in response to the spread of COVID-19 (which continued to threaten the health of participants and their families), participants had the opportunity to spend more time with their families, who may have previously received less attention.

“We have never had such long conversations, eaten all three meals, exercised, and played together like this before. My family has to be healthy together. Our familial love grew as we looked after one another. I’m trying to think of it as a gift from COVID-19.”

(Participant 4)

#### 3.4.2. Settling into Healthy Lifestyle Habits

The participants’ health was threatened by an irregular lifestyle and lack of exercise as they spent more time at home due to their inability to freely engage in outdoor activities. However, over time, they aimed to form physically and mentally healthy habits to maintain their health status in an environment that could not accommodate their desired level of exercise.

“We bought a bicycle for each of us and ride our bicycles together; we take the stairs instead of the elevator and try to walk instead of driving as much as possible. And I’m trying to live a healthy lifestyle by managing my stress and eating well-balanced meals for every meal.”

(Participant 7)

In addition, COVID-19 gave participants the opportunity to properly familiarize themselves with the concept of hygiene, which they had not been particularly interested in until then.

“My children used to wear a mask or wash their hands half-heartedly, but they do them well now. Moreover, there are hand sanitizers wherever you go—restaurants, banks, cafes, elevators, and more. The biggest change is the fact that products to prevent infection have permeated into our daily lives.”

(Participant 11)

#### 3.4.3. Actively Dreaming of and Preparing for a Healthy Future

With the occurrence of COVID-19, the participants realized the necessity of systematic education on infection prevention. They pondered methods to universalize health education, which had previously been neglected within Korea, and began to think of specific activities for infection prevention that could be practiced in people’s daily lives.

“It was hard to learn information other than what’s obvious—like wearing a mask, washing hands, and social distancing. If healthcare providers were to periodically educate residents on infection prevention at public health centers, wouldn’t it lead to actual infection control?”

(Participant 1)

Some participants emphasized that a national effort is needed to increase the number of negative-pressure rooms, which is currently deficient, and to reform the healthcare disparities between regions as revealed by the COVID-19 situation.

“My region has an insufficient number of negative-pressure rooms, and the overall condition of the medical facilities is poor. I felt that it is much more inadequate than that of Seoul, and policies are urgently needed to reform the healthcare system.”

(Participant 2)

## 4. Discussion

This study aimed to understand the life-changing experiences of mothers with school-age children during the spread of COVID-19, focusing on their health risk perceptions and health-related behaviors. The findings show that, as the participants gradually realized the true nature of COVID-19 through various information sources, they engaged in diverse efforts, in their role as mother and wife, to protect their families’ health. They would occasionally have a negative reaction to the seemingly endless pandemic but tried their best to adjust to the new lifestyle with COVID-19 by focusing on the management of their families’ health. This is similar to the idea proposed by Abdulkareem et al. [3], which states that an individual’s judgment of a situation and their resulting coping behaviors occur in accordance with their risk perceptions as formed by the information obtained from various sources during an epidemic.

First, participants gradually came to realize the true nature of COVID-19 through various sources of information. As they received a daily influx of COVID-19-related information through various channels, even if they did not want it—including SNS, television news programs, Internet news articles, SNS conversations with acquaintances, and so on—they began to feel a sense of fear and terror regarding the pandemic. The public generally acquires information related to infectious diseases through the media, the Internet, and social media [10]. The media’s reporting of disease-related news has a positive effect in that it can easily and quickly convey a large quantity of information regarding the disease and any necessary preventive measures. However, the media can also have an adverse effect because the reporting of inaccurate news, in addition to the indiscriminate quantity of information being reported, often fosters a sense of fear in the public [11,12]. Previous studies [10,11,12] have stated that media coverage of infectious diseases often conveys a sense of fear to the public, rather than simply providing people with disease-related information. They also state that, while the amount of online news articles consumed, the level of perceived fear, and the perception of others’ acquisition of information have a positive effect on individual hygienic behaviors, the fear-related perceptual bias has a significantly negative effect on peoples’ behavioral changes, which supports the results of this study. The participants of this study attempted to obtain accurate information using their investigative abilities to the fullest capacity after the health of their families was threatened, and the amount of indiscriminate information regarding COVID-19 intensified their anxiety levels. The sources of health-related information used most by women were television, radio, social media, and friends or acquaintances, which was consistent with the results of a previous study [4]. Therefore, platforms of mass media such as SNS, television news programs, and the Internet need to consider their effect on the public’s perception of the pandemic and people’s infection prevention behaviors that occur after the media conveys COVID-19 related information. Further, they should exercise caution in order to responsibly convey accurate information.

Second, participants became the person in charge of their family’s health and undertook various efforts to protect it. This is similar to the idea proposed by Abdulkareem et al. [3], which states that an individual’s judgment of a situation and their resulting coping behaviors occur in accordance with their risk perceptions as formed by the information obtained from various sources during an epidemic. Kim et al. [11] reported that the public uses the media, including mass media and the Internet, to process information from which they carry out sufficient reflective reasoning before forming risk perceptions and preventive behavioral intentions. The participants of this study perceived this pandemic as a threat based on the information they acquired and, as protectors of their families’ health, aimed to put into practice the health-related behaviors they could adopt to ensure their relatives’ health. They were active in pursuing items that their family members needed, including items for basic infection prevention, such as masks and hand sanitizers. They also prepared immune-boosting diets, dietary supplements, and functional foods, as well as handled the household’s laundry and cleaning. Some participants used methods of symptomatic therapy to strengthen their family members’ immune systems and prevent respiratory infections because they felt anxious about the lack of a specific treatment for COVID-19. However, caution must be exercised in the application of symptomatic therapies relating to the respiratory system, as there is a possibility that an infection may progress and worsen into an acute form of the disease without the administration of drug treatments at the appropriate time [13].

Third, participants reacted negatively to the seemingly endless COVID-19 situation. As the global number of confirmed cases has repeatedly risen and fallen, it has become increasingly difficult to predict the end of the COVID-19 pandemic. Schools have been closed, and various gatherings restricted [10], which has caused the participants of this study to feel frustrated. As a result, they spoke of their yearning for face-to-face human relationships as their daily lives consisted of contactless virtual systems, such as online education, online shopping, and so on. In order to solve this issue, recommendations have been made to revise the definition of “social distancing” to a more positive expression [10,14]. The WHO is changing the phrase “social distancing” to “physical distancing,” with researchers emphasizing that social solidarity would be strengthened when people realize the essential purpose of social distancing is to protect others, which ultimately protects themselves [14,15].

Fourth, participants tried to adjust to a new lifestyle during the COVID-19 pandemic. Experts predict that infections similar to COVID-19 will occur continuously in the future [10,16]. COVID-19 signifies more than just the emergence of a novel infectious disease; it is a historic event. In the future, time periods may be defined as occurring before or after the COVID-19 pandemic [17]. As the participants of this study experienced lifestyle changes resulting directly from COVID-19, they turned away from vague expectations that their past, normal lives would be restored and decided to adjust to this new reality. By experiencing multiple paradigm shifts in their lives firsthand—around not only healthcare services, but also the economy, society, culture, education, and so on—due to COVID-19, the participants accepted that their lifestyles would now have to adapt to this pandemic and tried their best to help their families live the healthiest lives possible. According to the findings of this study, the spread of COVID-19 was a negative event that threatened the participants’ health but also provided an opportunity to affirm their levels of familial love. It also served as an event through which they naturally learned about the concept of hygiene, realized the importance of exercise, and formed healthier lifestyle habits. In addition, they actively considered measures needed for living a healthy future life. These findings coincide with those of a previous study [18], which showed the development of behavioral changes in people to actively protect their health against COVID-19. In addition, the level of compliance with precautions against COVID-19 infections differed significantly between people depending on their age, gender, marital status, and level of education [5]. As the participants of this study were highly educated married women with an education level higher than a bachelor’s degree, it is assumed that they have a relatively high level of self-discipline for compliance with health-related behaviors aimed at protecting their families’ health. In order to prepare for the possible occurrence of future novel infections, the healthcare system should be updated, and strategies should be developed to invigorate public health education that utilizes schools and public health centers, thereby promoting Korean people’s prevention of infection as well as healthier lifestyle behaviors [19].

The study limitations and proposals for future research are as follows. First, as this study investigated the experiences of mothers with school-age children during the COVID-19 pandemic, its findings could differ depending on an individual’s stage of life, thus limiting generalizability. Second, as this study only reflected upon the viewpoint of mothers with school-age children, it was unable to determine the health risk perceptions and health-related behaviors experienced by fathers. Therefore, future studies should reflect upon the perceptions of fathers and explore the phenomena of health risk perceptions and health-related behaviors according to gender, as well as seek to understand these multifaceted phenomena as revealed by the family as a whole. Nevertheless, this study should be considered significant as it describes the life-changing experiences of Korean mothers, with a focus on their health risk perceptions and health-related behaviors caused by the global COVID-19 pandemic. In addition, this study reminds us of the importance of personal hygiene, which we theoretically knew about from the indiscriminate torrent of information in the context of the COVID-19 pandemic. It is meaningful to have confirmed that the importance has been put into practice and strengthened.

## 5. Conclusions

The findings of this study show that, while the participants were aware of COVID-19 through various sources of information and would at times become tired of the seemingly endless pandemic situation, they had a sense of duty as the mother of the family and tried their best to protect their families’ health. This study also determined that the participants were making an effort to adjust to their new lifestyles following the COVID-19 outbreak. Based on the findings of this study, the promotion of a safe media environment should take precedence through establishing a system to convey accurate information. This will ensure that the people of Korea will not be swayed by inaccurate news stories and will instead be able to recognize legitimate information. Furthermore, education on methods of infection prevention and a systematic health education system should be developed; prevention methods should be detailed and realistic, as well as relevant to people’s everyday lives, covering topics such as nutrition, physical activity, sleep, hygiene, stress management, and more, rather than merely emphasizing vague preventive measures, such as a basic code of conduct or social distancing. Future studies are needed to further verify the efficacy of these recommended measures.

## Figures and Tables

**Table 1:** Characteristics of the participants (*N =* 12).

No.	Age (Years)	Occupation	Number of Children	Location of Residence	Education
1	44	Homemaker	2	Incheon	University
2	36	Homemaker	2	Daegu	Master’s
3	39	Nurse	2	Seoul	Master’s
4	40	Nurse	2	Seoul	PhD
5	41	Homemaker	3	Seoul	University
6	36	Private business owner	2	Daegu	University
7	40	Private instructor	1	Daegu	Master’s
8	41	Homemaker	2	Incheon	University
9	42	Homemaker	2	Seoul	University
10	45	Homemaker	1	Seoul	University
11	44	Office worker	2	Gyeonggi-do	University
12	37	Homemaker	2	Gyeonggi-do	University

**Table 2:** List of interview questions.

Question Type	Questions
Introductory question	Please tell us about your overall life experiences changed by COVID-19.
Main questions	
Information source	How did you obtain information about COVID-19?
Perceived severity	How did you feel about accessing the media related to COVID-19? How did you feel about coming into contact with the COVID-19 epidemic area, work environment, and home environment? What health resources did my family and I need? What resources did I actually use, and which were scarce? How did you feel about hearing COVID-19-related content from people around you?
Perceived susceptibility	During the COVID-19 pandemic, were there any sick persons in your home, workplace, neighborhood, or school?
Perceived response efficacy	Please tell us about your health-related experiences/people around you during the COVID-19 pandemic.
Perceived self-efficacy	Have there been any changes in your economic condition or your role in the home?
Threat appraisal	What health threats have you felt during the COVID-19 pandemic? What health problems have you experienced during the COVID-19 pandemic? What were the obstacles to your health behavior?
Coping appraisal	How did you feel about experiencing the COVID-19 pandemic situation? What health behavior has changed? What infection control activities have you applied for yourself and your family? What kind of health care have you applied for your family and yourself? What are some of the facilitating factors you need for your family and your own health behavior?
Protection motivation	What have you done daily to improve your health during the COVID-19 pandemic? What is the difference between your current health promotion practices and those you practiced in your life before the COVID-19 pandemic? What kind of health behaviors do you really need for your family and yourself?
Ending statement	If you want to talk more about anything or have something else to add, please tell us.

**Table 3:** Life-changing experiences of mothers with school-age children during the COVID-19 pandemic (*N* = 12).

Themes	Sub-Themes	Codes
Struggling to identify the substance of COVID-19	Floundering in a deep swamp of confusion and fear	Not knowing the true nature of COVID-19
		Confusion caused by the spread of indiscriminate information
		Terrifying news on the media
		Life-threatening horror situation
	Desperately wanting reliable information	Doubtful about the information/Unfounded rumors
		Lack of knowledge in new infection situation
Taking the initiative to protect the health of the family	Devised methods for the prevention of infections	Various attempts to protect the health of the family
		Highlight washing hands
		Masks that are difficult to buy as usual
	Focus on family’s health management	Responsibility for family health
		Preparing healthy foods
		Encouraging exercise
	Attempted symptomatic therapy	Using unproven health care methods
		Listening to rumors to boost immunity
Frustrated by the brutal reality of no end in sight	Dissatisfaction with a lifestyle that has no way out	Endless spread of COVID-19 infections
		Feeling of exhaustion from repetitive routines
		Doubts about the end of COVID-19
	Yearning for people from within a dreary reality	Significant reduction in face-to-face opportunities
		Feeling stuffy about wearing a mask
		Our past is irreversible
Trying to adjust wisely to an inevitable new lifestyle	Strengthened family bonds	Spending a lot of time with my family
		Increased interest in family health
		Working out with family
	Settling into healthy lifestyle habits	Regular exercise
		Eating well-balanced meals
		Opportunity to learn about the concept of hygiene
	Actively dreaming of and preparing for a healthy future	Unavoidable global issue
		Needs for health education
		Needs for secure healthcare system

## Data Availability

The data presented in this study are available on request from the first author.
